# Fabrication of Surfactant-Enhanced Metal Oxides Catalyst for Catalytic Ozonation Ammonia in Water

**DOI:** 10.3390/ijerph15081654

**Published:** 2018-08-03

**Authors:** Chen Liu, Yunnen Chen, Lin Guo, Chang Li

**Affiliations:** Jiangxi Key Laboratory of Mining & Metallurgy Environmental Pollution Control, Jiangxi University of Science & Technology, Ganzhou 341000, China; chen_liu94@163.com (C.L.); GL1048020354@163.com (L.G.); lishuchang906@163.com (C.L.)

**Keywords:** catalytic ozonation, surfactant, metal oxide, ammonia nitrogen

## Abstract

The new surfactant-enhanced metal oxides composite catalysts have been prepared using solid state method and characterized by the N_2_-adsorption-desorption, scanning electron microscopy (SEM), energy dispersive X-ray spectroscopy (EDS), transmission electron microscope (TEM), and X-ray diffraction (XRD) techniques. Catalytic activity of the synthesized powders has been investigated in the liquid-phase catalytic ozonation ammonia nitrogen (NH_4_^+^) (50 mg/L). Especially, the effect of parameters such as optimum molar ratio for metal salt, NaOH and surfactants, temperature, and time of calcinations was also considered. Leveraging both high catalytic activity in NH_4_^+^ degradation and more harmless selectivity for gaseous nitrogen, the CTAB/NiO catalyst is the best among 24 tested catalysts, which was generated by calcining NiCl_2_·6H_2_O, NaOH, and CTAB under the molar ratio 1:2.1:0.155 at 300 °C for 2 h. With CTAB/NiO, NH_4_^+^ removal rate was 95.93% and gaseous nitrogen selectivity was 80.98%, under the conditions of a pH of 9, ozone flow of 12 mg/min, dosage of catalyst 1.0 g/L, reaction time 120 min, and magnetic stirring speed 600 r/min in room temperature.

## 1. Introduction

Over the past few decades, the nitrogen pollution in water has been increasing continuously due to various human activities, such as extensive use of fertilizers on agricultural landandNH_4_^+^-containing agents in the rare earth mineral extraction process [1,2]. Excessive NH_4_^+^ causes eutrophication of surface water, deterioration of the water quality, and the production of nitrates which endanger animals and human health [3,4]. Therefore, it is urgent to remove these contaminants from water. At present, there are many NH_4_^+^ wastewater treatment technologies, like biological nitrification, ion exchange, membrane separation, and break-point chlorination. Among them, the biological treatment method is stable but the reaction conditions need to be strictly controlled [5]. Ion exchange and membrane separation methods only convert pollutantreceptors [6,7]. Break-point chlorination causes the formation of chloramines, leading to secondary pollution [8]. Heterogeneous catalytic ozonation, where NH_4_^+^ can be removed as gaseous nitrogen by utilizing a catalyst to decompose ozone and produce hydroxyl radicals, has aroused considerable interest as a promising method [9,10]. Therefore, the key to heterogeneous catalytic ozonation technology is to find a kind of suitable catalyst.

In fact, various metal oxides, especially transition metal oxides with high redox and semiconductor properties, have been examined for use to improve the oxidizing capacity of ozone in catalytic ozonation of NH_4_^+^ in water [11]. Ichikawa et al. [12] studied a series of metal oxides catalysts’ (Fe_2_O_3_, CuO, Mn_3_O_4_, MgO, NiO, SnO_2_, Al_2_O_3_, Co_3_O_4_, ZnO) catalytic ozonation oxidation NH_4_^+^ at 60 °C and without pH control of the solution. It was confirmed that MgO and NiO have the highest activity but low selectivity to gaseous products, while Co_3_O_4_ has high selectivity to gaseous products but was slightly less active. On the other hand, NiO, Co_3_O_4_, and CuO are typical p-type oxide, while ZnO and MnO_2_ are typical n-type oxide. Bulanin et al. [13] proposed that the adsorption of O_3_ on the surface of n-type oxides result in the formation of surface-bound oxygen atoms (O). O_3_ reacts with p-type oxides to decompose O_3_, forming ionic intermediates ($O_{2}^{-}$↔O_2_^2−^) [13,14,15] with semi-peroxide, superoxide features, and triggering free-radical reactions (Equations (1)–(4)).In this process, both the generated hydroxyl radicals (·OH) and O have a stronger oxidation capacity than O_3_. The oxidation potentials of ·OH, ·O, and O_3_ are 2.80 V, 2.42 V and 2.07 V, respectively [16]. Therefore, in presence of ozone, these transition metal oxides as catalysts have the ability to degrade NH_4_^+^ in water. However, according to the previously mentioned [12], it is usually necessary to provide stronger reaction conditions (high temperature) due to the weak interaction between metal oxides and ozone.

(1) $$\equiv \mathrm{Me}-\mathrm{OH}+O_{3}\to \equiv \mathrm{Me}+O_{2}^{-}\cdot +{\mathrm{HO}}_{2}$$

(2) $$O_{2}^{-}\cdot +O_{3}\to O_{3}^{-}\cdot +O_{2}$$

(3) $$O_{3}^{-}\cdot +H^{+}\to {\mathrm{HO}}_{3}$$

(4) $${\mathrm{HO}}_{3}\cdot \to \cdot \mathrm{OH}+O_{2}$$

Recently, introduction of surfactants in the preparation of metal oxides has attracted much attention as a feasible and valuable method to control the surface properties of material, such as morphological structure, specific surface area, and particle size to improve the chemical reactivity. Leonardo et al. [17] reported that a flower-like CeO_2_ catalyst prepared with ethylene glycol using the co-precipitation method, exhibiting the best synergistic preferential oxidation activity, while Wu et al. [18] prepared CeO_2_ micron flowers using polyethylene glycol instead of ethylene glycol, which has a larger specific surface area and shows a kind of good activity to catalytic oxidation of CO. Additionally, the material obtained by this method can also be used as a gas sensor material [19] and applied to purify wastewater by decomposing organic compounds in water [20,21]. However, there are no similar and corresponding reports on the catalysts prepared by the combination of metal oxides and surfactant to enhance the interaction between catalyst and ozone for the catalytic ozonation of NH_4_^+^ in water.

In this study, surfactant-enhanced metal oxide catalysts have been prepared with different fabrication conditions using the solid state method and systematically evaluated. Three cationic surfactants and three anionic surfactants were used, which are commonly used and easily available. The prepared catalysts were characterized, and catalytic activity of the synthesized catalysts has been explored in the liquid-phase catalytic ozonationNH_4_^+^ (50 mg/L) as well.

## 2. Experimental

### 2.1. Materials

NH_4_Cl (Tianjin Damao Chemical Reagent Factory, Tianjin, China) was used to prepare simulated water containing NH_4_^+^. MnCl_2_·4H_2_O (Tianjin Damao Chemical Reagent Factory, Tianjin, China), NiCl_2_·6H_2_O, CuCl_2_·2H_2_O, ZnCl_2_ (Xirong Science Co. Ltd., Shanghai, China), CoCl_2_·6H_2_O (Shanghai Zhanyun Chemical Co. Ltd., Shanghai, China) were used for the preparation of metal oxides. Surfactants such as Cetyltrimethylammoniumbromide (CTAB), sodium dodecyl benzene sulfonate (SDBS), sodium dodecyl sulfate (SDS) (Sinopharm Chemical Reagent Co., Ltd., Shanghai, China), Cetyltrimethylammonium chloride (CTAC), benzalkoniumchloride (BAC), and sodium *N*-lauroylsarcosinate (SLS) (Shanghai Maclean Biochemical Technology Co., Ltd., Shanghai, China) were used for strengthening catalytic performance of metal oxides. All the materials were used without further purification. The distilled water was used for the preparation of all the catalysts and NH_4_^+^ solution.

### 2.2. Catalysts Preparation

Surfactant-enhanced metal oxide catalysts were prepared by a solid-state method. The solid of metal salt (CoCl_2_·6H_2_O, MnCl_2_·4H_2_O, NiCl_2_·6H_2_O, CuCl_2_·2H_2_O or ZnCl_2_), NaOH, and surfactants (CTAB, CTAC, BAC, SDBS, SDS or SLS) were mixed with the desired molar ratios, then placed in the agate mortar and ground for 60 min into a fine powder. After the solid mixture was washed several times repeatedly with distilled water, it was dried in an oven at 90 °C for 5 h, and subsequently milled and calcined in air at varying temperatures and periods. The prepared catalysts were named CTAB/Co_3_O_4_, CTAB/MnO_2_, CTAB/NiO, CTAB/CuO, CTAB/ZnO, CTAC/Co_3_O_4_, CTAC/MnO_2_, CTAC/NiO, CTAC/CuO, CTAC/ZnO, BAC/Co_3_O_4_, BAC/MnO_2_, BAC/NiO, BAC/CuO, BAC/ZnO, SDBS/Co_3_O_4_, SDBS/MnO_2_, SDBS/NiO, SDBS/CuO, SDBS/ZnO, SDS/Co_3_O_4_, SDS/MnO_2_, SDS/NiO, SDS/CuO, SDS/ZnO, SLS/Co_3_O_4_, SLS/MnO_2_, SLS/NiO, SLS/CuO, and SLS/ZnO.

For comparison, catalysts with only metal oxides but no surfactant were prepared under the same experimental procedures, which were named Co_3_O_4_, MnO_2_, NiO, CuO, and ZnO and were prepared similarly.

### 2.3. Catalysts Characterization

Samples for the specific surface areas were determined from N_2_ adsorption-desorption isotherm measurements at −196 °C according to the Brunauer-Emmett-Teller (BET) method. Morphologies of the samples were observed using scanning electron microscopy (SEM) and transmission electron microscopy (TEM). The crystalline structure was obtained using an X-ray diffraction meter (XRD) with Cu *K_α_* (λ = 1.540 Å, 40 kV, 40 mA) radiation.

### 2.4. Evaluation of Catalytic Activity

Catalytic ozonation experiments were carried out with a 250 mL reactor at room temperature (20 ± 2 °C). In a typical reaction, 200 mL of initial concentration 50 mg/L NH_4_Cl solution was introduced into the reactor. The initial pH value was adjusted to 9.0 with 2 M NaOH. 1 g/L catalyst was added into this solution under magnetic stirring in a stream of ozone (O_3_). O_3_ was generated by an ozone generator (FL-815ET, FeiLi, Shenzhen, China). The magnetic stirring speed was 600 r/min. Liquid samples were withdrawn from the reactor at given intervals and then the concentrationsof ammonia (NH_4_^+^), nitrite (NO_2_^–^), and nitrate (NO_3_^–^) in solution were measured.

### 2.5. Analyses and Calculations

The concentration of NH_4_^+^ ($C_{NH_{4}^{+}}$) and NO_2_^–^ ($C_{NO_{2}^{-}}$) in the liquid samples were measured by a visible spectrophotometer (SP-756PC, Shanghai Spectrum Instrument Co. Ltd., Shanghai, China) according to Nessler’s reagent spectrophotometry method [22] and spectrophotometry method [23], respectively. The nitrate ($C_{NO_{3}^{-}}$) concentration was measured by an ultraviolet spectrophotometer (722N, Shanghai Spectrum Instrument Co., Ltd., Shanghai, China) in accordance with the ultraviolet spectrophotometry method [24]. In this study, total nitrogen (TN) is composed of NH_4_^+^, NO_2_^–^, and NO_3_^–^ in solution and gaseous nitrogen. Percentages of NH_4_^+^ ($P_{NH_{4}^{+}}$), NO_3_^–^ ($P_{NO_{3}^{-}}$), and NO_2_^−^ ($P_{NO_{2}^{-}}$) were calculated by Equations (5)–(7):

(5) $$P_{NH_{4}^{+}}=\frac{C_{NH_{4}^{+}}}{C_{Initial\;NH_{4}^{+}}}\times 100\%$$

(6) $$P_{{NO}_{3}^{-}}=\frac{C_{NO_{3}^{-}}}{C_{Initial\;NH_{4}^{+}}}\times 100\%$$

(7) $$P_{{NO}_{2}^{-}}=\frac{C_{NO_{2}^{-}}}{C_{Initial\;NH_{4}^{+}}}\times 100\%$$

where $C_{NH_{4}^{+}}$ (mg/L), $C_{NO_{3}^{-}}$ (mg/L), and $C_{NO_{2}^{-}}$ (mg/L) are the final NH_4_^+^, NO_3_^—^, NO_2_^—^ concentrations in solution, respectively; $C_{Initial\;NH_{4}^{+}}$ (mg/L) represent the concentration of NH_4_^+^ in the liquid samples before reaction.

## 3. Results and Discussions

### 3.1. Catalytic Performances of Catalysts

**Figure 1:**
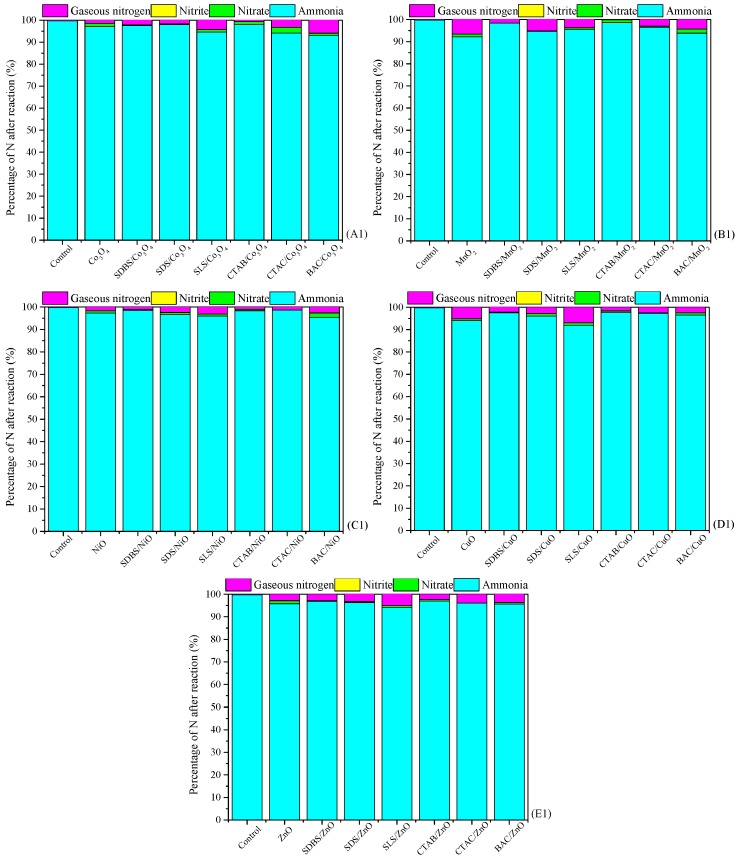
Conversion performance of the metal oxides and surfactant-enhanced metal oxides used for treatment of NH_4_^+^ in water. Catalyst preparation conditions: molar ratio of melt salt, NaOH and surfactant 1:2.1:0.093, calcined at 400 °C for 4 h; Reaction conditions: [NH_4_^+^], 50 mg/L from NH_4_Cl; initial pH of the solution, 9; dosage of catalyst, 1 g/L; magnetic stirring speed, 600 r/min; reaction temperature, 20 °C; and reaction time, 120 min. (**A1**) Conversion performance of control, Co_3_O_4_ and surfactant-enhanced Co_3_O_4_ catalysts used for treatment of NH_4_^+^ in water, (**B1**) Conversion performance of control, MnO_2_ and surfactant-enhanced MnO_2_ catalysts used for treatment of NH_4_^+^ in water, (**C1**) Conversion performance of control, NiO and surfactant-enhanced NiO catalysts used for treatment of NH_4_^+^ in water, (**D1**) Conversion performance of control, CuO and surfactant-enhanced CuO catalysts used for treatment of NH_4_^+^ in water, (**E1**) Conversion performance of control, ZnO and surfactant-enhanced ZnO catalysts used for treatment of NH_4_^+^ in water.

The conversion of NH_4_^+^ in the absence and presence of the metal oxides or surfactant-enhanced metal oxide without ozone is shown in Figure 1. In the control, NH_4_^+^ in solution did not decompose at all. In the presence of a catalyst, there was only a small amount of NH_4_^+^ that was absorbed and the removal rate was under 7%, indicating that the decomposition of NH_4_^+^ is not mainly due to the adsorption of NH_4_^+^ by the catalyst.

**Figure 2:**
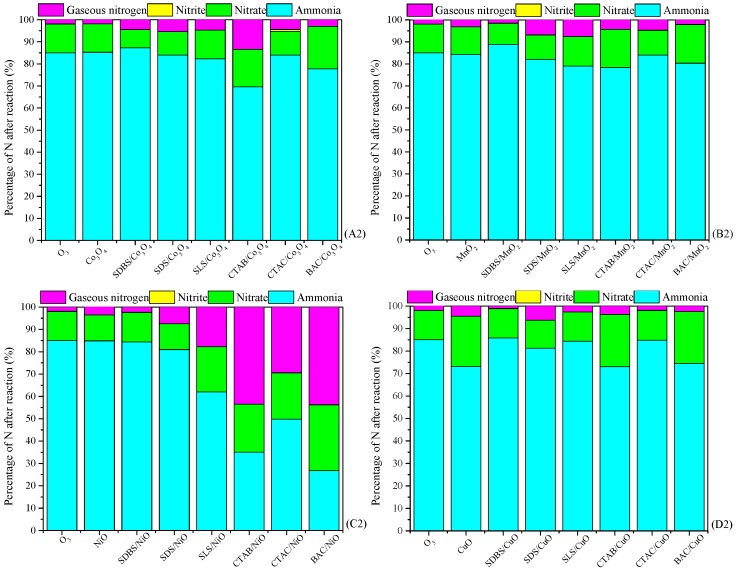

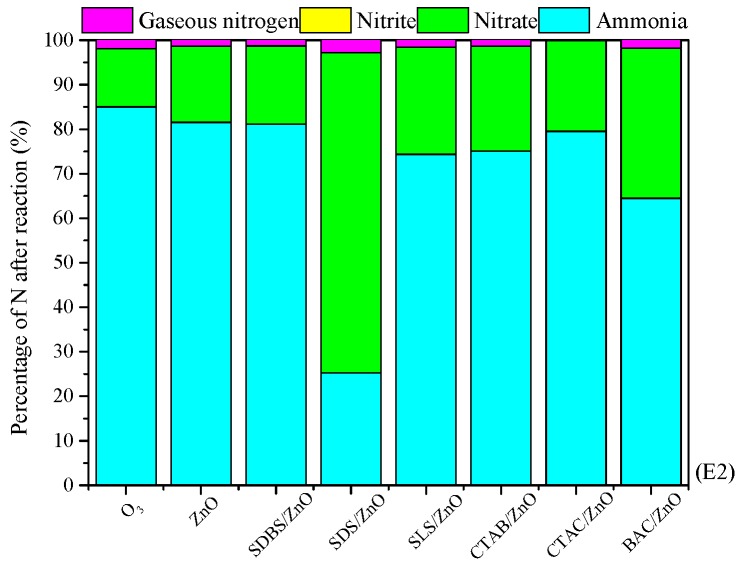
Catalytic performance of the metal oxide and surfactant-enhanced metal oxide for ozonation of NH_4_^+^ in water. Catalyst preparation condition: molar ratio of melt salt, NaOH and surfactant 1:2.1:0.093, calcined at 400 °C for 4 h; Reaction conditions: [NH_4_^+^], 50 mg/L from NH_4_Cl; initial pH of the solution, 9; dosage of catalyst, 1 g/L; magnetic stirring speed,600 r/min; ozone aeration rate, 12 mg/min; reaction temperature, 20 °C; and reaction time, 120 min. (**A2**) Catalytic performance of O_3_, Co_3_O_4_ and surfactant-enhanced Co_3_O_4_ catalysts used for treatment of NH_4_^+^ in water, (**B2**) Catalytic performance of O_3_, MnO_2_ and surfactant-enhanced MnO_2_ catalysts used for treatment of NH_4_^+^ in water, (**C2**) Catalytic performance of O_3_, NiO and surfactant-enhanced NiO catalysts used for treatment of NH_4_^+^ in water, (**D2**) Catalytic performance of O_3_, CuO and surfactant-enhanced CuO catalysts used for treatment of NH_4_^+^ in water, (**E2**) Catalytic performance of O_3_, ZnO and surfactant-enhanced ZnO catalysts used for treatment of NH_4_^+^ in water.

As a comparison, catalytic performance of metal oxides and surfactant-enhanced metal oxides used for ozonation of NH_4_^+^ in water is shown in Figure 2. In the case of ozone without catalyst, the removal rate of NH_4_^+^ was about 15% and most of the removed NH_4_^+^ was converted to NO_3_^–^ (13%), which is more hazardous than NH_4_^+^. In the case of metal oxide catalysts without ozone, their catalytic performance is almost negligible. As for anionic surfactant-enhanced metal oxide catalysts, SDS/ZnO can greatly reduce the NH_4_^+^ content in the solution, but it produced a large amount of undesired species of NO_3_^–^. Among cationic surfactant-modified metal oxide catalysts, the ozonation of NH_4_^+^ catalyzed by BAC/NiO contributed to the lowest residual NH_4_^+^ (26.74%), but the highest NO_3_^–^ (29.52%). There was no significant difference in the content of NO_3_^–^ produced by the ozonation of NH_4_^+^ with CTAB/NiO and CTAC/NiO (respectively 21.46% and 20.77%), but the residual NH_4_^+^ content was lower than the latter, respectively 35.06% and 49.78%. Based on the high removal rate of NH_4_^+^ and the low conversion of NO_3_^–^, it can be concluded that CTAB/NiO is the catalyst with the best catalytic performance.

**Figure 3:**
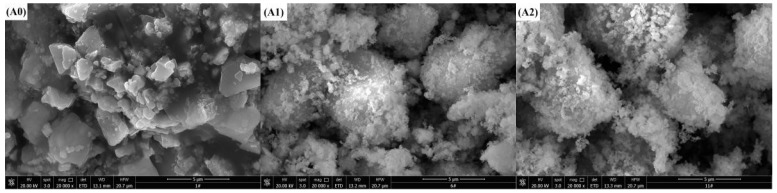

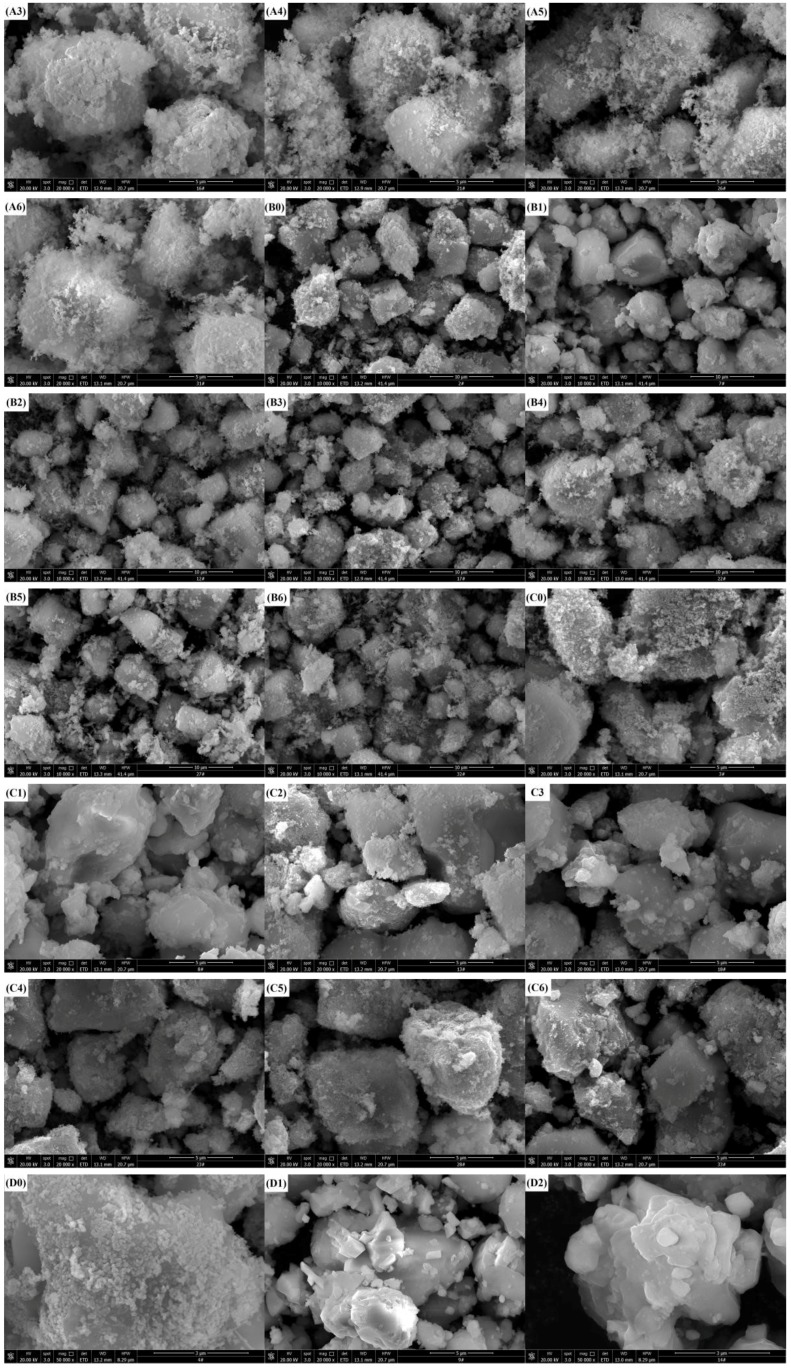

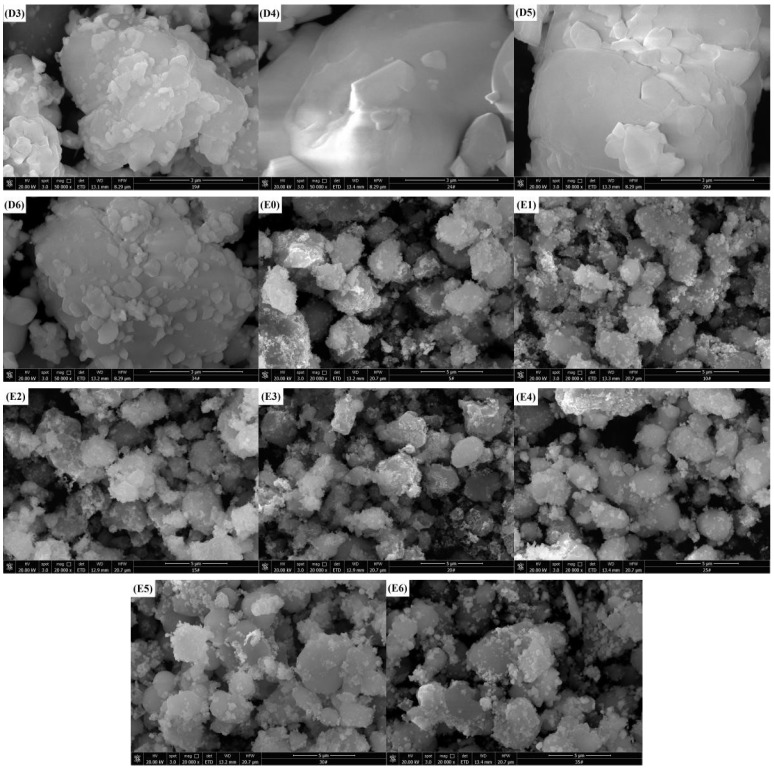
Scanning electron microscopy (SEM) images of catalysts. (**A0**) Co_3_O_4_, (**A1**) SDBS/Co_3_O_4_, (**A2**) SDS/Co_3_O_4_, (**A3**) SLS/Co_3_O_4_, (**A4**) CTAB/Co_3_O_4_, (**A5**) CTAC/Co_3_O_4_, (**A6**) BAC/Co_3_O_4_; (**B0**) MnO_2_, (**B1**) SDBS/MnO_2_, (**B2**) SDS/MnO_2_, (**B3**) SLS/MnO_2_, (**B4**) CTAB/MnO_2_, (**B5**) CTAC/MnO_2_, (**B6**) BAC/MnO_2_; (**C0**) NiO, (**C1**) SDBS/NiO, (**C2**) SDS/NiO, (**C3**) SLS/NiO, (**C4**) CTAB/NiO, (**C5**) CTAC/NiO, (**C6**) BAC/NiO; (**D0**) CuO, (**D1**) SDBS/CuO, (**D2**) SDS/CuO, (**D3**) SLS/CuO, (**D4**) CTAB/CuO, (**D5**) CTAC/CuO, (**D6**) BAC/CuO; (**E0**) ZnO, (**E1**) SDBS/ZnO, (**E2**) SDS/ZnO, (**E3**) SLS/ZnO, (**E4**) CTAB/ZnO, (**E5**) CTAC/ZnO, (**E6**) BAC/ZnO.

The surface components of solid catalysts were usually complicated and unevenly distributed. The surface structure that participated in catalytic reaction had many active sites with a special physical structure. Figure 3 shows SEM micrographs of the different types of catalysts. Figure 3A0 reveals Co_3_O_4_ in the form of flakes and small pieces, while the Co_3_O_4_ after the addition of the surfactant is irregularly bulky and has a large number of small particles on the surface, as shown in Figure 3A1–A6. MnO_2_ shown in Figure 3B0 is a pebble-like block with a superficially thick surface. Figure 3B1 indicates that the surface of bulk MnO_2_ particle becomes flat and smooth with the addition of SDBS. From Figure 3B2–B6, the morphology of SDS/MnO_2_, SLS/MnO_2_, CTAB/MnO_2_, CTAC/MnO_2_, and BAC/MnO_2_ has no significant difference from that of MnO_2_. Figure 3C0 shows the surface of NiO has a large amount of homogeneous and small particles, while Figure 3C1 with SDBS/NiO becomes smooth. The morphology of SDS/NiO, SLS/NiO, CTAB/NiO, CTAC/NiO, and BAC/NiO (Figure 3C2–C6) has no remarkable difference from that of NiO. Figure 3D0 reveals that the surface of CuO has distributed with a large amount of irregular and small particles. While the surface of surfactant-modified CuO has not distribute densely with particles. The surface of ZnO has large amount of small and compact blocky particles as shown in Figure 3E0. Figure 3E1,E6 display that the surface of SDBS/ZnO and BAC/ZnO samples has some sparse spherical particles, while that of SDS/ZnO, SLS/ZnO, CTAB/ZnO, and CTAC/ZnO has no significantly sparse spherical particles, as shown in Figure 3E2–E5.

### 3.2. Optimizations of Fabrication Conditions for CTAB/NiO Catalyst

#### 3.2.1. Effect of Surfactant Addition on Catalytic Performance

**Figure 4:**
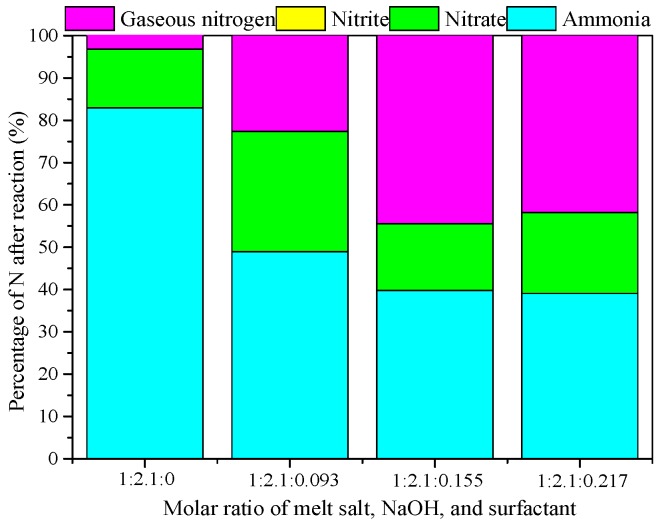
Effect of molar ratio on catalytic performance. Catalyst preparation condition:molar ratio of melt salt, NaOH and surfactant from 1:2.1:0 to 1:2.1:0.217, calcined at 400 °C for 4 h; Reaction conditions: [NH_4_^+^], 50 mg/L from NH_4_Cl; initial pH of the solution, 9; dosage of catalyst, 1 g/L; magnetic stirring speed,600 r/min; ozone aeration rate, 12 mg/min; reaction temperature, 20 °C; and reaction time, 120 min.

The amount of added surfactant may affect the surface chemical properties of the catalyst during the preparation process. On the other hand, it may influence on the physical structure characteristics and the activity of the catalyst. From Figure 4, it can be seen that the residual NH_4_^+^ in the solution decreased with increasing content of surfactant in the catalyst, while the percentage of gaseous nitrogen increased. With molar ratio of melt salt, NaOH and surfactant 1:2.1:0 to 1:2.1:0.093, the existing CTAB was not efficient to let O_3_ adsorb on the catalyst surface and decomposed to produce oxide species such as OH and O, thereby further degrading the NH_4_^+^ to reach the best result. When the molar ratio increased to 1:2.1:0.155, the content of gaseous nitrogen selectivity was the highest, which was 44.44%, with a NH_4_^+^ removal rate of 60.23%.

#### 3.2.2. Effect of Calcination Temperature on Catalytic Performance

**Figure 5:**
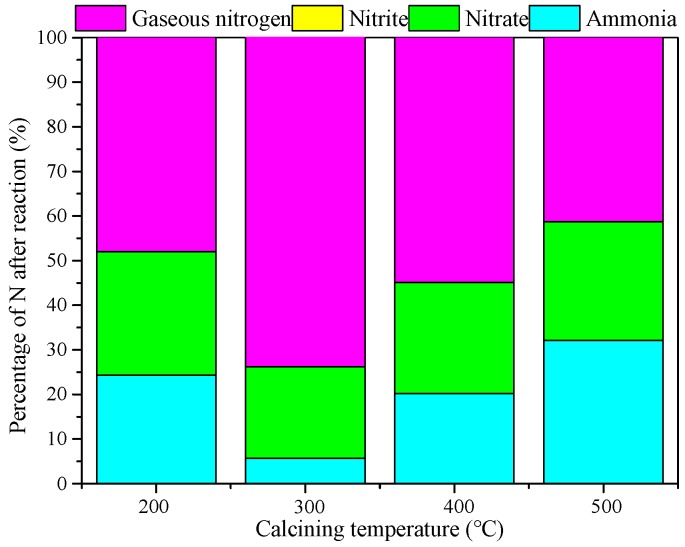
Effect of Calcination temperature on catalytic performance. Catalyst preparation conditions: molar ratio of melt salt, NaOH and surfactant 1:2.1:0.155, calcined from 200 to 500 °C for 4 h; Reaction conditions: [NH_4_^+^], 50 mg/L from NH_4_Cl; initial pH of the solution, 9; dosage of catalyst, 1 g/L; magnetic stirring speed, 600 r/min; ozone aeration rate, 12 mg/min; reaction temperature, 20 °C; and reaction time, 120 min.

The calcination temperature might affect the surface properties of the catalyst. Figure 5 shows that with the calcining temperature increasing, the residual NH_4_^+^ in solution decreased first and then increased slightly while the content of gaseous nitrogen had the opposite trend. When the temperature rose up to 300 °C, the content of NH_4_^+^ was the lowest, at 5.68%, and gaseous nitrogen selectivity was 73.79%. Possibly when the calcination temperature is higher than a certain threshold, the crystallinity of CTAB/NiO strengthened and the number of crystal lattice defects reduced, and both resulted in fewer active sites. Therefore, the activity of the catalyst tended to decrease when the temperature exceeded 300 °C.

#### 3.2.3. Effect of Calcination Time on Catalytic Performance

**Figure 6:**
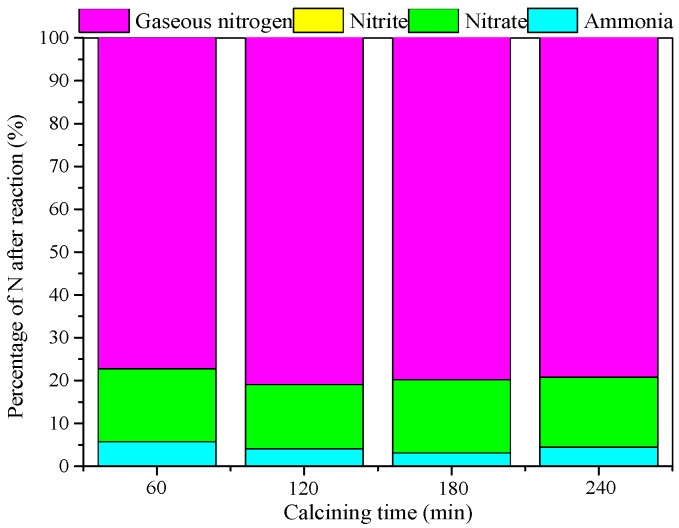
Effect of calcination time on catalytic performance. Catalyst preparation condition: molar ratio of melt salt, NaOH and surfactant 1:2.1:0.155, calcined at 300 °C for 1–4 h; Reaction conditions: [NH_4_^+^], 50 mg/L from NH_4_Cl; initial pH of the solution, 9; dosage of catalyst, 1 g/L; magnetic stirring speed, 600 r/min; ozone aeration rate, 12 mg/min; reaction temperature, 20 °C; and reaction time, 120 min.

Figure 6 shows that the NH_4_^+^ removal was 95.93% and gaseous nitrogen selectivity was 80.98% when the calcination time was 120 min. However, the NO_3_^−^ content increased with continuous increasing time in the calcination. The particles tended to agglomerate possibly due to sintering of the catalyst after being calcined for too long, which led to the active sites on the catalyst surface reduce.

### 3.3. Characterizations of CTAB/NiO Catalysts

The specific surface areas of the catalysts with different molar ratios, different temperatures, and times of calcinations are shown in Table 1. It can be seen that the specific surface area of NiOwas 7.77 m^2^/g. For the CTAB/NiO series, the specific surface areas decrease with increasing surfactant content because of the increase of surface coverage by the surfactant particles prevents the entry of nitrogen probe molecules. In addition, when the molar ratio of NiCl_2_·6H_2_O, NaOH and CTAB was 1:2.1:0.155, the specific surface area increased first and then decreased with different temperatures and times of calcination. One possible reason is some of the surfactants doped in the catalyst were partially lost during calcination, so the catalyst formed a pore structure and the calcination temperature was too high to make the catalyst sintered. The calcination time had little effect on the specific surface area of the catalyst with molar ratio 1:2.1:0.155, while the maximum specific surface area was19.45 m^2^/g when the catalyst calcined at 300 °C for 2 h.

**Figure 7:**
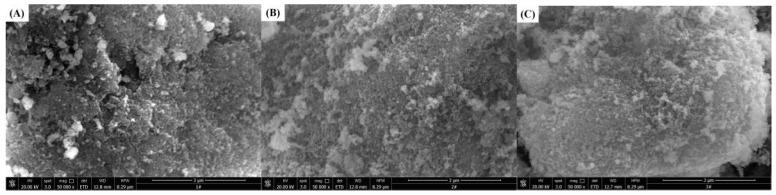

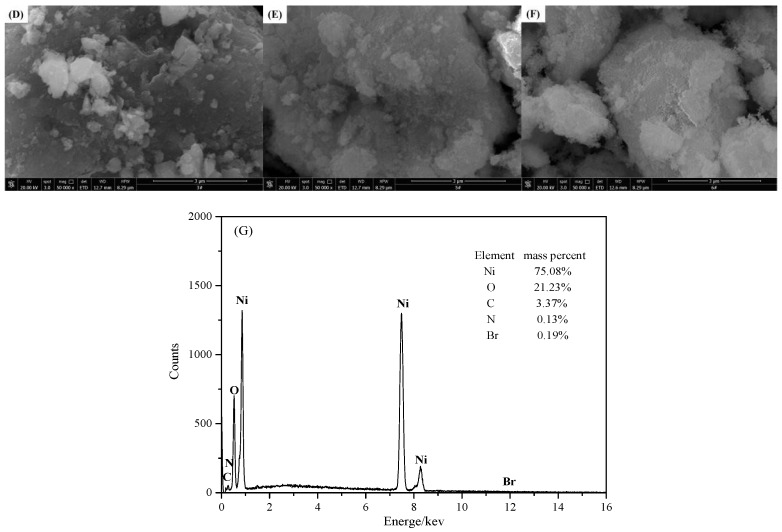
SEM images of catalysts. (**A**) CTAB/NiO-1, (**B**) CTAB/NiO-2, (**C**) CTAB/NiO-3, (**D**) CTAB/NiO-4, (**E**) CTAB/NiO-5, (**F**) CTAB/NiO-6, (**G**) EDS images of CTAB/NiO-2 (inset: mass percentages of Ni, O, C, N and Br).

**Figure 8:**
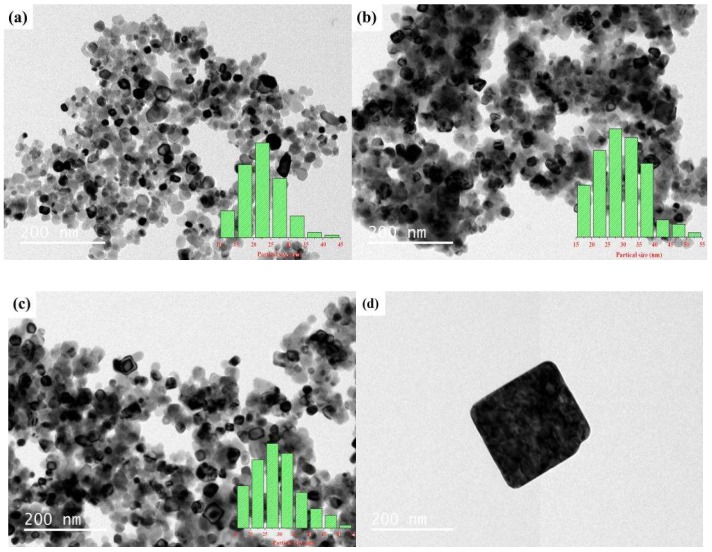
Transmission electron microscopy (TEM) images of catalysts: (**a**) CTAB/NiO-1, (**b**) CTAB/NiO-2, (**c**) CTAB/NiO-3, (**d**) CTAB/NiO-2.

For the CTAB/NiO catalysts with a molar ratio of metal salt, NaOH, and surfactant 1:2.1:0.155, there were no significant variance in the morphology of the catalysts at different calcinating temperatures for 4 h, as shown in Figure 7A–C. However, Figure 7D–F showed that the on surface of the catalyst a large amount of spherical small particles was gradually distributed with the elapse of calcinating time under 300 °C. Figure 7G shows a SEM-energy dispersive X-ray spectroscopy (EDS) image of the CTAB/NiO-2 catalyst. The EDS analysis indicates the presence of Ni, O, C, N, and Br on the CTAB/NiO-2 surface. The atomic percentage of Ni and O is about 1:1 based on the mass percentages, which indicates that the metal oxide in the catalyst may be NiO. The TEM images revealed hexagonal and tetragonal plate-like structure under CTAB/NiO-1, CTAB/NiO-2, and CTAB/NiO-3 (Figure 8). However, with CTAB content increasing, tetragonal plate-like structures were more dominant, which indicated that the morphological structure of the catalyst has a transition from hexagonal to square. Figure 8D shows a square CTAB/NiO-2 sample. According to the TEM images, the average particle size in CTAB/NiO-1, CTAB/NiO-2 and CTAB/NiO-3 samples is 22.65, 29.23, and 29.20 nm, respectively.

**Figure 9:**
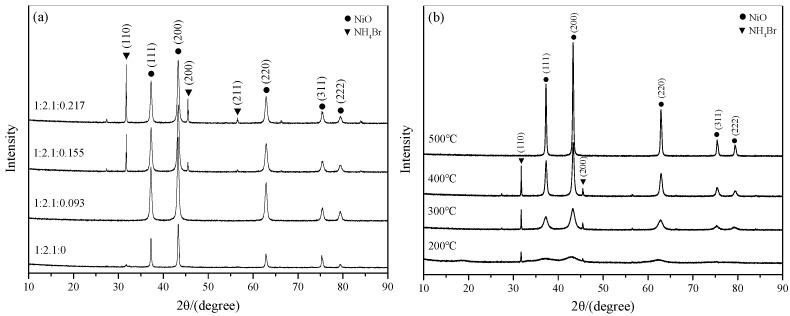
X-Ray diffraction (XRD) pattern of catalysts. (**a**) CTAB/NiO catalysts with different molar ratios, calcined at 400 °C for 4 h, (**b**) CTAB/NiO catalysts with molar ratios 1:2.1:0.155, calcined at different temperatures for 4 h.

Figure 9 shows the X-ray diffractometer (XRD) patterns of the CTAB/NiO catalysts with different molar ratios, calcined at different temperatures for 4 h. From the XRD pattern, diffraction peaks can be indexed as NiO (JCPDS 47–1049) and NH_4_Br (JCPDS 73–1493). From Figure 9a it can be seen that no NH_4_Br was detected at the molar ratio 1:2.1:0.155. The potential reason is that the NH_4_Br formed under this condition is less and dispersed in the catalyst. While the peak height of NH_4_Br increased by the increasing of the amount of CTAB in the catalyst. On the other hand, Figure 9b indicates that the thermal treatment has a significant effect on the catalyst. The peak of NiO becomes sharper and narrower with the increasing of the calcination temperature, illustrating the crystallinity of NiO particles was enhanced and the purity was higher. When the temperature was increased from 200 °C to 400 °C, the peak intensity of the NH_4_Br component in the catalyst strengthened gradually. When the temperature was to 500 °C, the peak of NH_4_Br disappeared. It may be due to the fact that CTAB decomposes gradually to produce NH_4_Br as calcination temperature increasing, and its peak disappears because of the excessive temperature leading to thermal decomposition of NH_4_Br. Leverage the experimental results from the Section 3.2.1 and Section 3.2.2, the active component of NH_4_Br in the catalyst may play an important role in catalytic ozonation ammonia nitrogen in water.

## 4. Conclusions

Surfactant-enhanced metal oxides composite catalysts fabricated by a solid-state method were used in the catalytic degradation of NH_4_^+^ under in presence of ozone. The catalysts were analyzed using BET, SEM, EDS, TEM, and XRD. The SEM and the specific surface area suggested that CTAB/NiO has a rough surface. The addition of CTAB improved the specific surface area of NiO, which can provide more active sites for catalytic reactions. The EDS analysis indicates the atomic percentage of Ni and O of the CTAB/NiO-2 catalyst is about 1:1 based on the mass percentages, which indicates that the metal oxide in the catalyst may be NiO. The TEM images of the CTAB/NiO catalysts indicated that the morphological structure of the catalyst has a transition from hexagonal to square, when CTAB content increased. Considering the experimental results and the XRD analysis, the active component of NH_4_Br in the catalyst may play an important role in catalytic ozonation of NH_4_^+^ in water. In terms of high catalytic activity in NH_4_^+^ degradation and gaseous nitrogen selectivity, the CTAB/NiO catalyst with NiCl_2_·6H_2_O, NaOH and CTAB molar ratio 1:2.1:0.155, calcined at 300 °C for 2 h, is the best one among the catalyst we tested, with an NH_4_^+^ removal rate of 95.93% and gaseous nitrogen selectivity was 80.98%. Therefore, surfactant-enhanced metal oxides catalyst is considered as a feasible method to improve the harmless degradation of NH_4_^+^.

## 5. Future Research Directions

According to the calculation of nitrogen balance, the products of catalytic ozonation oxidation are gaseous nitrogen in addition to the residual NH_4_^+^, NO_3_^−^, and NO_2_^–^ in the solution. The gaseous nitrogen in this study may contain NH_3_, N_2_, NO_2_, and N_2_O etc. In our follow-up experiment we will analyze qualitatively and quantitatively various substances present in gaseous nitrogen. At the same time, another issue worth considering is the catalyst recycling. In summary, it is important to make surfactant-enhanced metal oxide catalysts feasible for efficient water purification and sewage treatment application so future study needs to address these issues mentioned above.

## Figures and Tables

**Table 1:** The specific surface areas of various catalysts.

Catalyst	Molar Ratio of Metal Salt, NaOH and Surfactant	Calcining Temperature (°C)	Calcining Time (h)	S_BET_ (m^2^/g)
NiO	1:2.1:0	400	4	7.77
CTAB/NiO-1	1:2.1:0.093	400	4	12.94
CTAB/NiO-2	1:2.1:0.155	400	4	7.96
CTAB/NiO-3	1:2.1:0.217	400	4	6.15
CTAB/NiO-4	1:2.1:0.155	200	4	5.48
CTAB/NiO-5	1:2.1:0.155	300	4	11.47
CTAB/NiO-6	1:2.1:0.155	500	4	3.71
CTAB/NiO-7	1:2.1:0.155	300	1	16.08
CTAB/NiO-8	1:2.1:0.155	300	2	19.45
CTAB/NiO-9	1:2.1:0.155	300	3	16.22

## References

[1] Zhou Y., Lv P., Dou Y.M. (2012). Advances in rare earth ammonia nitrogen wastewater treatment technology. Nor. Environ..

[2] Ibendahl G., Fleming R.A. (2007). Controlling aquifer nitrogen levels when fertilizing crops: A study of groundwater contamination and denitrification. Ecol. Model..

[3] Torrento C., Cama J., Urmeneta J., Otero N., Soler A. (2010). Denitrification of groundwater with pyrite and Thiobacillusdenitrificans. Chem. Geol..

[4] Schwientek M., Einsiedl F., Stichler W., Stogbauer A., Strauss H., Maloszewski P. (2008). Evidence for denitrification regulated by pyrite oxidation in a heterogeneous porous groundwater system. Chem. Geol..

[5] Waki M., Tokutomi T., Yokoyama H., Yokoyama H., Yasuo T. (2007). Nitrogen removal from animal waste treatment water by Anammox enrichment. Bioresource Technol..

[6] Tilaki R., Kahe D. (2012). Advances in Environment, Biotechnology and Biomedicine.

[7] Rezakazemi S., Shirazian S., Ashrafizadeh S.N. (2012). Simulation of ammonia removal from industrial wastewater streams by means of a hollow-fiber membrane contactor. Desalination.

[8] Lee J.K., Lee K.R., Hong S.H., Kim K.H., Lee B.H., Lim J.H. (2002). Residual chlorine distribution and disinfection during electrochemical removal of dilute ammonia from an aqueous solution. J. Chem. Eng. Jpn..

[9] Haag W.H., Hoigne J., Bader H. (1984). Improved ammonia oxidation by ozone in presence of bromide ion during water treatment. Water Res..

[10] Chen Y.N., Wu Y., Liu C., Guo L., Nie J., Chen Y., Qiu T.S. (2018). Low-temperature conversion of ammonia to nitrogen in water with ozone over composite metal oxide catalyst. J. Environ. Sci..

[11] Nawrocki J., Kasprzyk-Hordem B. (2010). The efficiency and mechanisms of catalytic ozonation. Appl. Catal. B Environ..

[12] Ichikawa S.I., Mahardiani L., Kamiya Y. (2014). Catalytic oxidation of ammonium ion in water with ozone over metal oxide catalysts. Catal. Today.

[13] Bulanin K.M., Lavalley J.C., Tsyganenko A.A. (1995). Infrared study of ozone adsorption on TiO_2_ (Anatase). J. Phys. Chem..

[14] Li W., Gibbs G.V., Oyama S.T. (1998). Mechanism of ozone decomposition on a manganese oxide catalyst. 1. In situ Raman spectroscopy and Ab initio molecular orbital calculations. J. Am. Chem. Soc..

[15] Naydenov A., Stoyanova R., Mehandjiev D. (1995). Ozone decomposition and CO oxidation on CeO_2_. J. Mol. Catal. A Chem..

[16] Liu Y., He H.P., Wu D.L., Zhang Y.L. (2016). Heterogeneous catalytic ozonation reaction mechanism. Prog. Chem..

[17] Peiretti L.F., Tiscornia I.S., Miró E.E. (2013). Study of the synthesis of CeO_2_nanoparticies for their use in CO preferential oxidation (COPrOx). Chem. Eng. J..

[18] Wu H.J., Wang L.D. (2011). Shape effect of microstructured CeO_2_ with Various morphologies on CO catalytic oxidation. Catal. Commun..

[19] Zhang G.H., Wang P.Y., Deng X.Y., Chen Y., Gengzang D.J., Wang X.L., Chen W.J. (2016). CTAB-assisted synthesis of 3D Sn doped ZnO nanostructures with enhanced acetone sensing performance. Mater. Lett..

[20] Begum S., Ahmaruzzaman M. (2018). CTAB and SDS assisted facile fabrication of SnO_2_nanoparticles for effective degradation of carbamazepine from aqueous phase: A systematic and comparative study of their degradation performance. Water Res..

[21] Sinirtas E., Isleyen M., Soylu G.S.P. (2016). Photocatalytic degradation of 2,4-dichlorophenol with V_2_O_5_-TiO_2_ catalysts: Effect of catalyst support and surfactant additives. Chin. J. Catal..

[22] Water Quality―Determination of Ammonia Nitrogen―Nessler’s Reagent Spectrophotometry. HJ 535-2009. http://kjs.mep.gov.cn/hjbhbz/bzwb/jcffbz/201001/t20100112_184155.shtml.

[23] Water Quality—Determination of Nitrogen (Nitrite)-Spectrophotometric Method. GB 7493-87. http://kjs.mep.gov.cn/hjbhbz/bzwb/jcffbz/198708/t19870801_66628.shtml.

[24] Water Quality—Determination of Nitrate-Nitrogen—Ultraviolet Spectrophotometry. HJ/T 346-2007. http://kjs.mep.gov.cn/hjbhbz/bzwb/jcffbz/200703/t20070316_101688.shtml.

